# Filling the Roots of Second Molars

**Published:** 1893-02

**Authors:** 


ARTICLE II.

### FILLING THE ROOTS OF SECOND MOLARS.

The following question has been sent us, with the request that it be answered in these pages:

"If a superior second molar, with pulpitis and pericementitis to the extent of soreness is presented, and you decide to destroy and remove the pulp and fill the roots, how would you proceed in detail, the patient being in good general health and the mouth in fair condition?"

The second molar presents but few complications that are not found in the first. It is further back in the mouth, and hence a little more difficult of access, but the roots or pulp chambers do not differ materially. The first symptom that demands attention is the pulpitis, for the pericementitis is dependent upon that. If there be considerable congestion of the pulp, it sometimes is not easy to destroy it without considerable pain. Usually, however, this will yield when the cavity of decay, which we will assume is the cause, has been cleaned out, and the pulp thoroughly exposed and bled. If not, tincture of aconite will give relief.

Of course it makes a great deal of difference where the cavity of decay is. If it is in the occluding, or mesial surface, there will be little trouble. If it is upon the distal aspect, the task will demand more care and skill. In either case, the cavity should be thoroughly rinsed with warm water, using repeated douches. With enamel chisels the cavity should be opened up so as to allow free access. The rubber dam should be applied, and the cavity dried out with the hot air blast. Usually this will obtund the tissues sufficiently to allow working without severe pain. If it does not, carbolic acid may be used, and the decayed tissue and debris should then be as thoroughly removed as possible. All pain will by this time usually have ceased.

If it has, the pulpitis will give no further trouble. If not, medication with aconite may be necessary. If there be excessive tenderness in the living pulp, this may be overcome by applying a solution of cocaine.

I know of no better devitalizing application than that recommended by Dr. Miller, in the July number of this journal. It consists of equal parts of arsenious acid and cocaine hydro-chlorate, mixed into a paste with a sufficient quantity of carbolic acid. A little—a very little—of this should be placed in a minute cup of tin or lead, prepared as follows: From a sheet of taggers tin, or rolled tin, or lead, punch out, or with the shears cut out, a disk of a sufficient size to cover the bottom of the cavity. Place it upon a piece of soft wood, and with the rounded end of an excavator handle indent it until it has a cup-shaped depression. Put the arsenious acid mixture in this, and carry it to place over the exposed pulp.

My usual way is to cover this with a pledget of cotton dipped in chloro-percha. Miller recommends the oxy-sulphate of zinc, or even plaster of paris, and these no doubt have their advantages. But by the use of the protecting disk, pressure upon the exposed pulp by a cotton plug may be avoided. I leave this application in the cavity about forty-eight hours. It may probably be left longer without danger, but at the end of two days I usually find the pulp thoroughly devitalized, and without much sensation.

At the end of this time the dressing is removed, using the rubber dam as before. The pulp chamber is opened thoroughly, and an antiseptic introduced. This may be one of the essential oils, or it may be our old and much abused, yet excellent friend, carbolic acid. I propose to coagulate the ends of the dentinal fibrillæ, if it has not already been done, which is most probable. The cavity is then sealed up again for another two days, when it is once more opened under the same precautions as before. A delicate Donaldson barbed broach is now introduced into the root canals, turned a little and withdrawn, usually with the pulp clinging to it. I do this for each of the three roots—if I can—then introduce pledgets of cotton upon a delicate smooth broach, carrying them as near to the apex of the roots as possible, and again seal the cavity up, unless there be some urgent necessity for haste.

When I am ready for filling the roots, I do it with chloro-percha, pumped into each with a smooth broach. When I am satisfied the root canal is full, I introduce a gutta percha point, and that root is supposed to be effectually sealed for all time.

I said that I go to the apex of each of the three roots if I can. Unfortunately this is not always practicable. I may state it stronger than that, and say that it is not often possible. The conformation of the roots will not permit. There is usually but little difficulty in finding and reaching the apex of the lingual root, but the other two are not as accessible. In the first place, both the anterior and posterior buccal roots are frequently curved, the flexure usually being forward. This makes the anterior root canal hard to find and follow, even if the opening be patulous. If the cavity of decay be upon the distal surface, it is absolutely essential that it be extended up through the coronal surface, quite to or past the central pit. The openings of all the roots must positively be uncovered.

If with a delicate broach I find that I cannot penetrate either canal, or for but a short distance, I seal the cavity up with gutta-percha, first introducing into it a small pledget of cotton wet with an antiseptic, and leave it for a week or ten days. By this time the contents of that canal will have sloughed away, or at the least will have separated from all attachments, and will come away of its own accord, without putrefaction. I can then treat and fill the canals as well as possible. If they are too small to admit the most delicate broach, I have not much fear of their containing sufficient matter, even though it have putrescent possibilities, to cause any harm. I cannot believe that the contents of the dentinal tubuli ever become septic. The tubuli are too small to permit the entrance of putrefactive organisms, or if not, the amount of putrescible matter is not sufficient to produce septic complications.

To a much less extent is this true of those root canals that are too small for the entrance of a fine broach. If time enough be given for the sloughing of the canal contents, and if then they are subjected to the penetrating action of an antiseptic, and finally if the mouth of the canal be hermetically sealed with gutta-percha, I will take my chances with it. There is, in fact, nothing else to do, for my wildest flights of imagination do not reach to the point of successfully drilling on such roots as these, with any possible kind of a drill.

The posterior buccal root has even another possible complication. It is apt to be wide and flat, and very thin in the center, the canal having the same characteristics. At either lateral border of the root there may be a channel, while in the center it is very much constricted, or even obliterated. There may be chambers somewhat along the course of the canal, which it will be utterly impossible thoroughly to clean out. There is absolutely no way of removing any pulp tissue from them, except by the slow process of allowing it to disintegrate and slough out, antiseptics excluded, for they would tend to prevent this. If the pulp chamber be open, this may sometimes be permissible, trusting to the penetrative power of antiseptics thoroughly to cure them afterward.

But with the utmost care and thoroughness there will be cases presented in which it is quite impossible to be sure of the condition of the roots. At least I find it so in my practice, for I am not one of those who can see to the bottom of an impenetrable root canal. But with thorough antiseptic treatment, I have little fear of any subsequent trouble from such roots.

It may be urged that all this is a very tedious and prolonged process. Well, if the dentist is not prepared to give to the case all the time and attention necessary, he had better not undertake it. The proper treatment and filling of the roots of a superior second molar tooth is a process that requires care, and patience, and skill, and he who does not possess enough of the last two to insure the first, can never take rank as a successful operator.—EDITORIAL in *Dental Pract. and Advertiser.*

